# A functional approach to understanding the role of NCKX5 in Xenopus pigmentation

**DOI:** 10.1371/journal.pone.0180465

**Published:** 2017-07-10

**Authors:** Ruth M. Williams, Robert J. Winkfein, Rebecca S. Ginger, Martin R. Green, Paul P. Schnetkamp, Grant N. Wheeler

**Affiliations:** 1 School of Biological Sciences, University of East Anglia, Norwich, United Kingdom; 2 Department of Physiology and Pharmacology, Hotchkiss Brain Institute, Cumming School of Medicine, University of Calgary, Calgary, Canada; 3 Unilever Research and Development, Colworth Science Park, Sharnbrook, Bedfordshire, United Kingdom; Simon Fraser University, CANADA

## Abstract

NCKX5 is an ion exchanger expressed mostly in pigment cells; however, the functional role for this protein in melanogenesis is not clear. A variant allele of SLC24A5, the gene encoding NCKX5, has been shown to correlate with lighter skin pigmentation in humans, indicating a key role for SLC24A5 in determining human skin colour. SLC24A5 expression has been found to be elevated in melanoma. Knockdown analyses have shown SLC24A5 to be important for pigmentation, but to date the function of this ion exchanger in melanogenesis has not been fully established. Our data suggest NCKX5 may have an alternative activity that is key to its role in the regulation of pigmentation. Here *Xenopus laevis* is employed as an *in vivo* model system to further investigate the function of NCKX5 in pigmentation. SLC24A5 is expressed in the melanophores as they differentiate from the neural crest and develop in the RPE of the eye. Morpholino knockdown and rescue experiments were designed to elucidate key residues and regions of the NCKX5 protein. Unilateral morpholino injection at the 2 cell stage resulted in a reduction of pigmentation in the eye and epidermis of one lateral side of the tadpole. *Xenopus* and human SLC24A5 can rescue the morpholino effects. Further rescue experiments including the use of ion exchange inactive SLC24A5 constructs raise the possibility that full ion exchanger function of NCKX5 may not be required for rescue of pigmentation.

## Introduction

Pigmentation is the natural colouration of animal tissue, it provides camouflage and protection, and offers a means of communication. Melanin is responsible for the majority of pigmentation seen in animals, synthesized in specialised organelles termed melanosomes found within pigment cells—melanocytes in humans and melanophores in fish and frogs. Melanin pigmentation is the main form of protection against UV induced DNA damage [1]. Many pathologies are associated with defects in pigmentation pathways including; vitiligo, albinism and melanoma [2–4].

SLC24A5 was first identified in the zebrafish as the gene responsible for the golden pigmentation phenotype. A mutation in the gene led to a truncated mutant protein which causes a significant reduction in pigmentation [5]. A non-synonymous SNP (single nucleotide polymorphism), identified in the human gene [6], encodes an amino acid switch (alanine to threonine) at position 111 of the protein. The two different alleles have been shown to be population specific. Ala111 is found at 93–100% in African, East Asian and indigenous American populations, whereas Thr111 is found in 98.7–100% of European and American populations [5–8].

SLC24A5 encodes NCKX5, a member of the NCKX family of potassium dependent sodium calcium exchangers [9, 10]. There are 4 other NCKX transmembrane proteins (1–4), which extrude calcium across the plasma membrane using the sodium potassium gradient. NCKX proteins are found in a variety of tissues, but their roles have not yet been fully elucidated. NCKX1 provides the only calcium extrusion pathway in retinal rod cells [11]. NCKX2 is found in the photoreceptor cone cells and throughout the brain [12, 13]. NCKX3 is also widely expressed in the brain, although it is also found in smooth muscle tissues [14]. NCKX4 is also detected in the brain, particularly the hippocampus as well as other tissues including heart, stomach and kidney [15–17]. NCKX5 is almost exclusively expressed in melanocytes, studies so far have shown NCKX5 to be partially co-localised to the trans Golgi network, [18], unlike other NCKX proteins which are thought to be active in the plasma membrane [19, 20]. This intracellular localisation is particularly intriguing and suggests a role for this exchanger in maintaining organelle ionic concentrations. The location of the protein makes it very difficult to perform functional analysis, however some data have been obtained. Using an insect high five cell expression system NCKX5 has been shown to transport Ca^2+^ across the membrane, but is less efficient in this system than its relative NCKX2. The variant allele of NCKX5 was found to be less active than the wild type [18]. NCKX2 has been the subject of numerous biochemical studies; these have revealed key regions and residues of the protein which are crucial to its ion exchanger function.

Knockdown of SLC24A5 using siRNA’s in human and murine melanocytes, *in vitro*, results in a significant decrease in pigmentation [18] and treatment with siRNA’s to SLC24A5 also resulted in a decrease in protein levels of several known melanosome markers; Pmel17, MART1, Tyr and Tyrp1, but an increase in protein levels of the lysosome marker Lamp1. Gene microarray analysis on siRNA NCKX5 treated normal human melanocytes also showed a loss of MCR1 and altered expression of a number of cholesterol homeostatic genes [21]. Together this data indicates NCKX5 is involved in early melanosome formation. Knockout mice displayed hypopigmentation of the IPE and RPE suggesting a role for SLC24A5 in ocular albinism [22]. Recent studies have also linked mutations in the SLC24A5 gene to Non-Syndromic Oculocutaneous Albinism (OCA6) [23]; [24].

*Xenopus laevis* has previously been demonstrated as a useful tool for examining pigment phenotypes [25] and was selected for further analysis of SLC24A5. This work presents an *in vivo* study of SLC24A5 and aims to further elucidate the function of this gene product (NCKX) in pigmentation.

## Results

### Cloning of SLC24A5

No previous work has been conducted on *X*.*laevis* SLC24A5 and no sequence data were available. Therefore bioinformatic approaches including NCBI and ensembl searches were employed to investigate the sequence information available from other species. Sequence data was found for human, mouse, zebrafish and *Xenopus tropicalis* and when aligned together the amino acid sequences have an overall similarity of 68.7%. Crucially mouse, zebrafish and *X*.*tropicalis*, all carry the ancestral alanine residue at the equivalent amino acid position 111. The *Xenopus tropicalis* sequence (ensembl no. ENSXETG00000018406) was used to design PCR primers. These primers were used with *X*.*laevis* cDNA to generate an 800bp fragment of SLC24A5, this was followed up with RACE PCR to generate the full length sequence. This shows 70% similarity to the protein sequence from other species. *X*.*laevis* and *X*.*tropicalis* NCKX5 are 84.7% similar (S1 Fig). At the equivalent amino acid 111 position of the human sequence, an alanine residue is present, indicating *X*.*laevis* does not carry the ns-SNP observed in humans. The *X*.*laevis* SLC24A5 open reading frame is 1.5kb in length, encoding a protein of approximately 50kDa. Subsequent to this work the *X*. *laevis* genome has become available. We have identified both long (L) and short (S) homologs of SLC24A5 found at chr3L:93,768,908–93,788,695 and chr3S:49,373,429–49,395,845 respectively. Our full length clone corresponds to the L form.

### SLC24A5 is expressed in pigment cells

**Fig 1:**
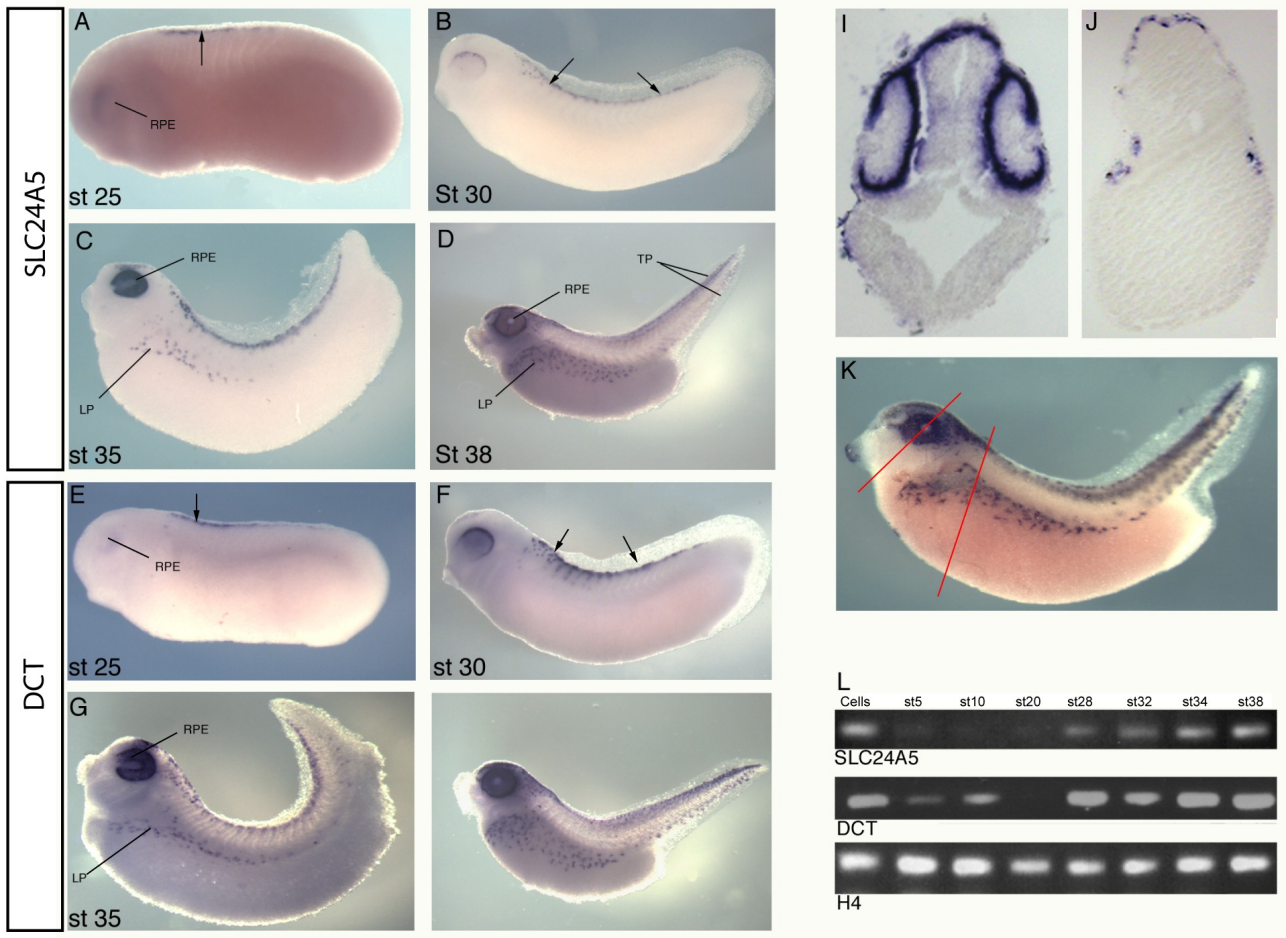
Expression of SLC24A5 in *X*.*laevis*. (A-K) Whole mount in situ hybridisation, (L) RT-PCR. (A-D) Expression of SLC24A5. (A) Expression initiates in the neural crest (arrow) and RPE at stage 25. (B) Expression continues in the melanophores as they mature from the neural crest as indicated by arrows. (C) Expression continues in the melanophores in the lateral pigmentation (LP) and increases in the RPE. (D) By stage 38 strong expression can be seen in melanophores of the lateral pigmentation, tail pigmentation (TP) and RPE. (E-H) Expression of the melanophore marker DCT in *X*.*laevis*. (E) Expression in the arising neural crest is indicated by arrow, (F) DCT is expressed in the melanophores as they mature from the neural crest as indicated by arrows. (G) and (H) Expression is maintained as melanophores migrate into lateral and tail pigmentation, expression continues in the RPE of the eye as indicated. (I-K) Histological analysis of SLC24A5 expression. (I) Section through the head reveals restricted expression to the RPE and dorsal head. (J) Section through the trunk shows expression in the flanks of the tadpole trunk and head. (K) whole embryo before sectioning, red line indicate approximate area where above sections were taken. (L) RT PCR analysis of SLC24A5 from various stages of *X*.*laevis* development. DCT was used a melanophores marker comparison, H4, loading control. Cells used were *X*.*laevis* melanophores (kind gift from Vladimir Gelfand).

To determine the expression pattern of SLC24A5 in *X*.*laevis*, whole mount *in situ* hybridisation and RT PCR analysis were conducted. An RNA probe for *in situ* analysis was synthesised from the newly obtained sequence data. Expression was first seen in stage 25 embryos in the eye and in the early melanophores (Fig 1A). At stage 30 expression increases as more melanophores develop from the neural crest (Fig 1B). At stage 35 expression can be seen in the melanophores of the lateral pigmentation, and boldly in the eye (Fig 1C). By stage 38, when maximum pigmentation is present, expression of SLC24A5 is detected throughout the expanded lateral pigmentation and the pigment cells of the tail, dorsal and ventral, and in the RPE (retinal pigmented epithelium) of the eye. The expression pattern of SLC24A5 correlates well to that of the known melanophore marker DCT, (Fig 1). To further analyse the expression pattern of SLC24A5 *in situ* hybridised embryos were processed for sectioning (Fig 1I–1K). Here it is clear the SLC24A5 expression in the eye is restricted to the RPE, while there is also expression in the dorsal head region. Lateral expression is restricted to the epidermal layer consistent with the location of melanophores.

SLC24A5 expression was also analysed by RT PCR (Fig 1L). Detection was observed at stage 28 through to stage 35 onwards. This is consistent with the expression of DCT although maternal DCT is also expressed at earlier stages.

### Loss of SLC24A5 causes a lack of pigment

Using the full length sequence of *X*.*laevis* SLC24A5 a morpholino was designed to the 5’ ATG translational start site. Sequence analysis shows that the morpholino corresponds to both the L and S forms of SLC24A5. To show the morpholino was targeting SLC24A5, *in vitro* translation reactions were carried out. The morpholino blocked SLC24A5 translation and did not block translation of controls (S2 Fig).

**Fig 2:**
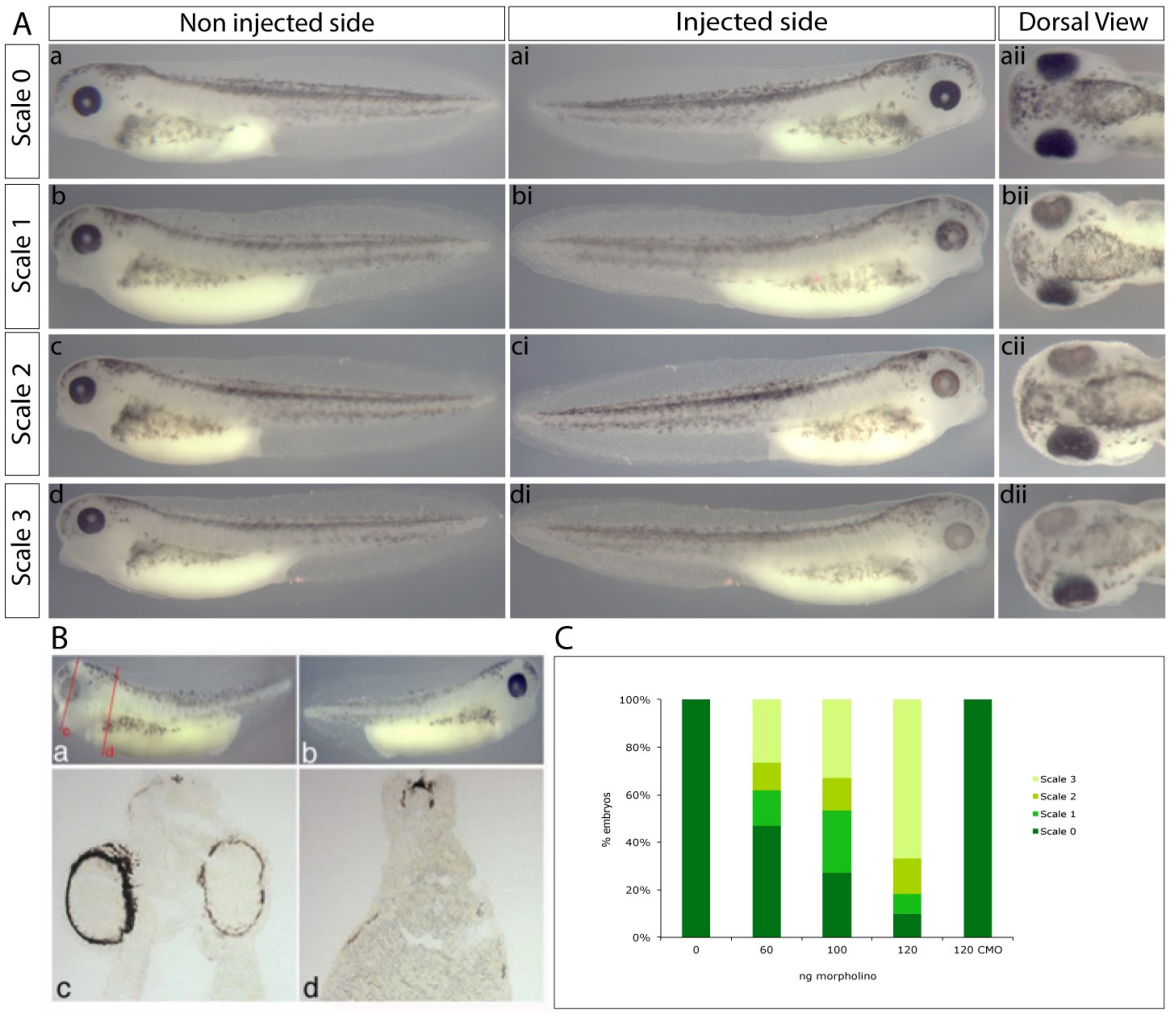
Morpholino knockdown of SLC24A5. (A) Scale of morpholino effect, a, ai, aii, scale 0 no effect; b, bi, bii, scale 1 subtle effect; c, ci, cii, scale 2 clear effect; d, di, dii, scale 3 obvious effect. a-d non injected control sides, ai-di injected sides showing effect, aii-dii dorsal view of embryos in a-d and ai-di highlighting relative effects in the eye. (B) Cryosectioning of morpholino treated embryos, a injected side; b non injected side; c and d sections of embryo in a and b, relative locations of sections are shown on a. (C) Dose response of morpholino, CMO control morpholino.

The morpholino was injected into 1 cell at the 2-cell stage at various concentrations and resulted in a reduction of pigmentation in the eye and lateral pigment in the injected side of the tadpole, with the non-injected side providing a negative control. A scale was devised to qualify the results, which varied in severity, as shown in Fig 2A a-d. Scale 0 represents tadpoles where the morpholino had no effect (Fig 2A a-aii). Scale 1 showed a subtle effect predominantly in the eye (Fig 2A b-bii). Scale 2 tadpoles had a more clear effect in the eye and the lateral pigmentation was slightly affected (Fig 2A c-cii). Scale 3 is the most obvious effect where pigmentation was almost lost in the eye and significantly reduced in the lateral pigmentation (Fig 2A d-dii). Increasing the concentration of morpholino caused an increase in the percentage of tadpoles with a more severe pigmentation effect, while the scrambled control morpholino had no effect (Fig 2C). Similar pigmentation phenotypes were observed using a splice site morpholino targeting the last intron/exon boundary (S4 Fig).

To further characterise the morpholino knockdown sections were taken of tadpoles following morpholino treatment (Fig 2B). This highlights the reduction of pigmentation in the RPE and lateral pigmentation.

**Fig 3:**
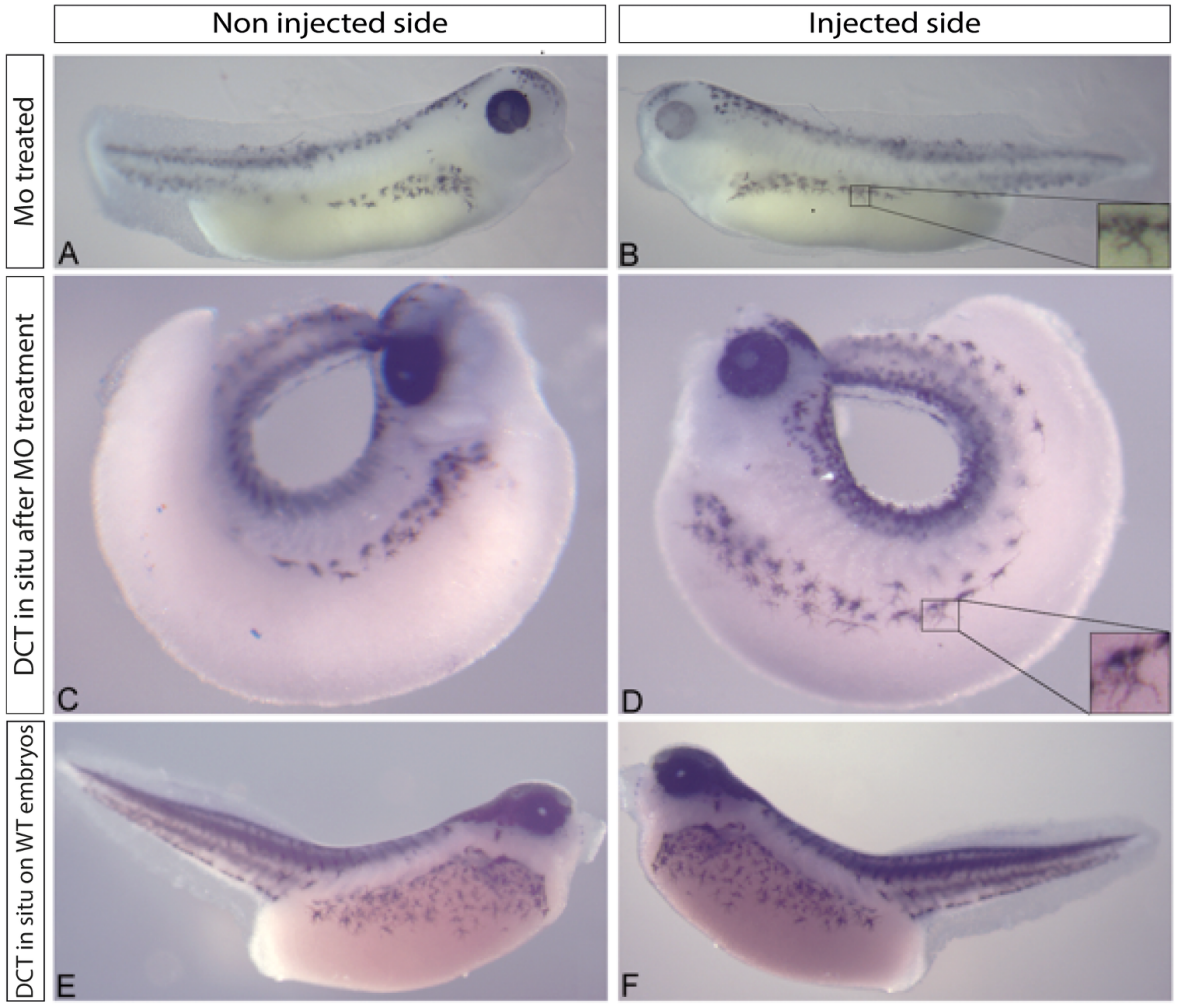
Expression of DCT following morpholino knockdown of SLC24A5. (A) Non injected side. (B) 100ng morpholino injected side. (C) Embryo in (A) following DCT in situ. (D) Embryo in (B) following DCT in situ. (E,F) Non injected controls following DCT in situ, n = 19.

It has previously been shown that siRNA mediated repression of SLC24A5 results in a decrease in protein levels of several pigmentation genes. We used *in situ* hybridisation following morpholino treatment to determine if the transcription of candidate pigment genes were affected by the morpholino treatment. Neither DCT (Fig 3), nor tyrosinase (data not shown) expression was affected by morpholino knockdown of SLC24A5.

### Morpholino mediated loss of NCKX5 can be rescued with WT and mutant constructs of NCKX5

To further investigate the function of NCKX5 in pigmentation we carried out rescue experiments by co-injecting the morpholino with mutant human SLC24A5 constructs to determine the crucial regions and residues of the protein. NCKX proteins are transmembrane proteins and are generally comprised of an N-terminal cleavable signal peptide followed by a short extracellular loop joined to 2 clusters of 5 transmembrane domains that are separated by a large cytoplasmic loop [26].

**Fig 4:**
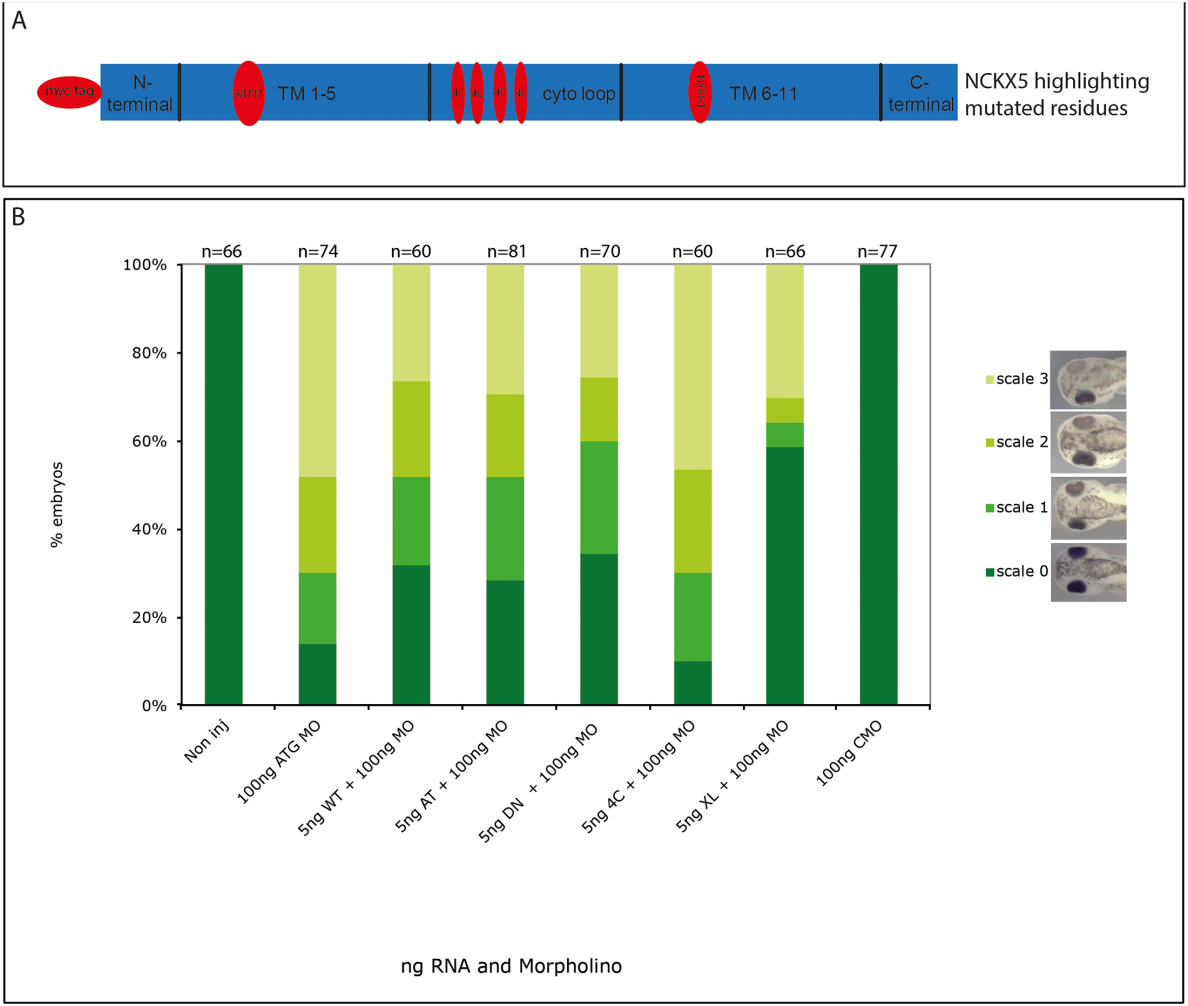
Rescuing the morpholino phenotype. Embryos were injected with 100ng morpholino and 5ng cRNA of the various constructs. Embryos were allowed to develop to stage 38. The experiments were repeated in triplicate and the results were combined, n values shown at top of graph. (A) schematic of rescue constructs. (B) Embryos scored for pigmentation effect as scale in Fig 2. NI non injected, WT wild type, AT A111T, DN D383N, XL *X*.*laevis*, mo morpholino, CMO control morpholino. Kruskal Wallis test revealed significant difference between 100ng ATG MO and the WT, AT, DN and XL constructs, p = 0.001 for all, but not 4C p = 0.988.

Initial rescue experiments were performed with the wild type human construct, the A111T variant allele, and two mutants based on mutational analysis of the related protein NCKX2. The first mutant (DN) has a D383N change, which is equivalent to D548N in NCKX2 [27]. D548 is suggested to be one of two key Ca^2+^ coordinating residues and neutralizing the charge in the D548N substitution completely abolished Ca^2+^ transport function (Kang et al 2005). Therefore, the equivalent D383N substitution in NCKX5 is expected to yield a mutant NCKX protein unable to transport Ca^2+^ across the membrane and was not expected to rescue the morpholino phenotype. In the second mutant (4C), a highly conserved cluster of 4 cysteine residues in the cytoplasmic loop have been replaced with glycine. This construct was used to determine the importance of the cysteine residues in the loop, which are unique to NCXK5. A mutant *X*.*laevis* construct was also developed where the morpholino target site was mutated such that the morpholino would not recognise it. Schematic of rescue constructs are shown in Fig 4.

100ng of morpholino caused a knockdown such that only 13.7% of embryos are scale 0 (no effect) (Fig 4A). This proportion increased significantly to 31.6% when the wild type human construct is co-injected representing a partial rescue (Kruskal Wallis test p<0.05). The A111T and D383N constructs also resulted in a significant partial rescue, where scale 0 embryos are increased to 28.4% and 34.2% respectively (Kruskal Wallis test p<0.05). Only the 4C construct could not rescue the effect of the morpholino. The *X*.*laevis* construct gave the strongest rescue, increasing the scale 0 embryos to 58.3%. No effect was seen when RNA of the rescue constructs was injected alone indicating the absence of an over-expression phenotype. The morpholino does not target the human SLC24A5 sequence, (S2 Fig) and all rescue constructs could be detected by western blotting with an anti myc antibody, following injection in the embryo (S3 Fig).

**Fig 5:**
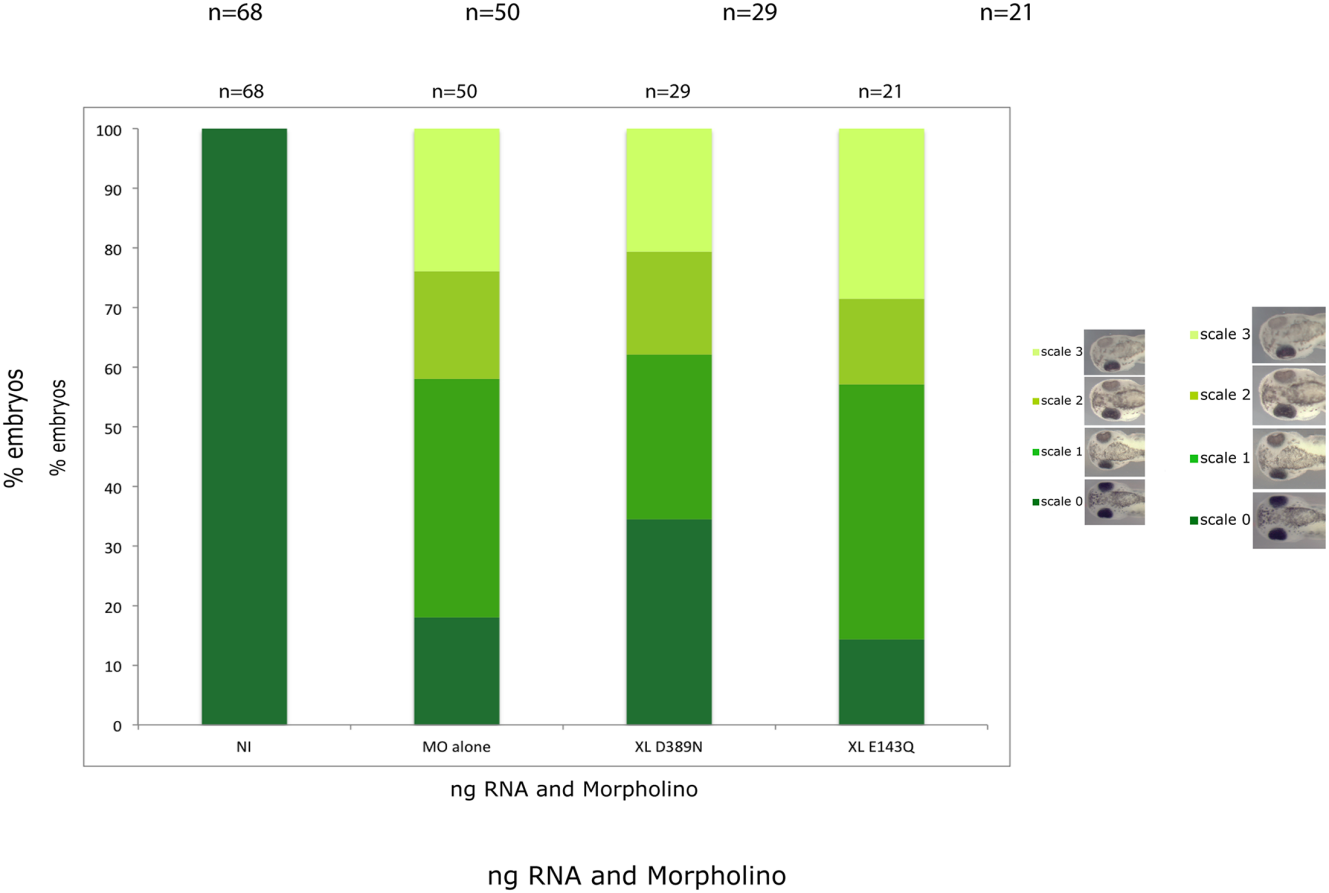
Rescuing with Xenopus mutants. Embryos were injected with 100ng morpholino and 5ng cRNA of the mutant constructs. Embryos were allowed to develop to stage 38. The experiments were repeated in triplicate and the results were combined, n values shown at top of graph.

To confirm the rescue results with the human constructs, we translated the D383N mutant into the Xenopus gene (D389N) and used this alongside another mutant E143Q to assess rescue capability. As shown in Fig 5, D389N can provide a partial rescue, consistent with our observations with the human construct. However, the Xenopus E143Q mutant cannot rescue.

### Chimera rescues

**Fig 6:**
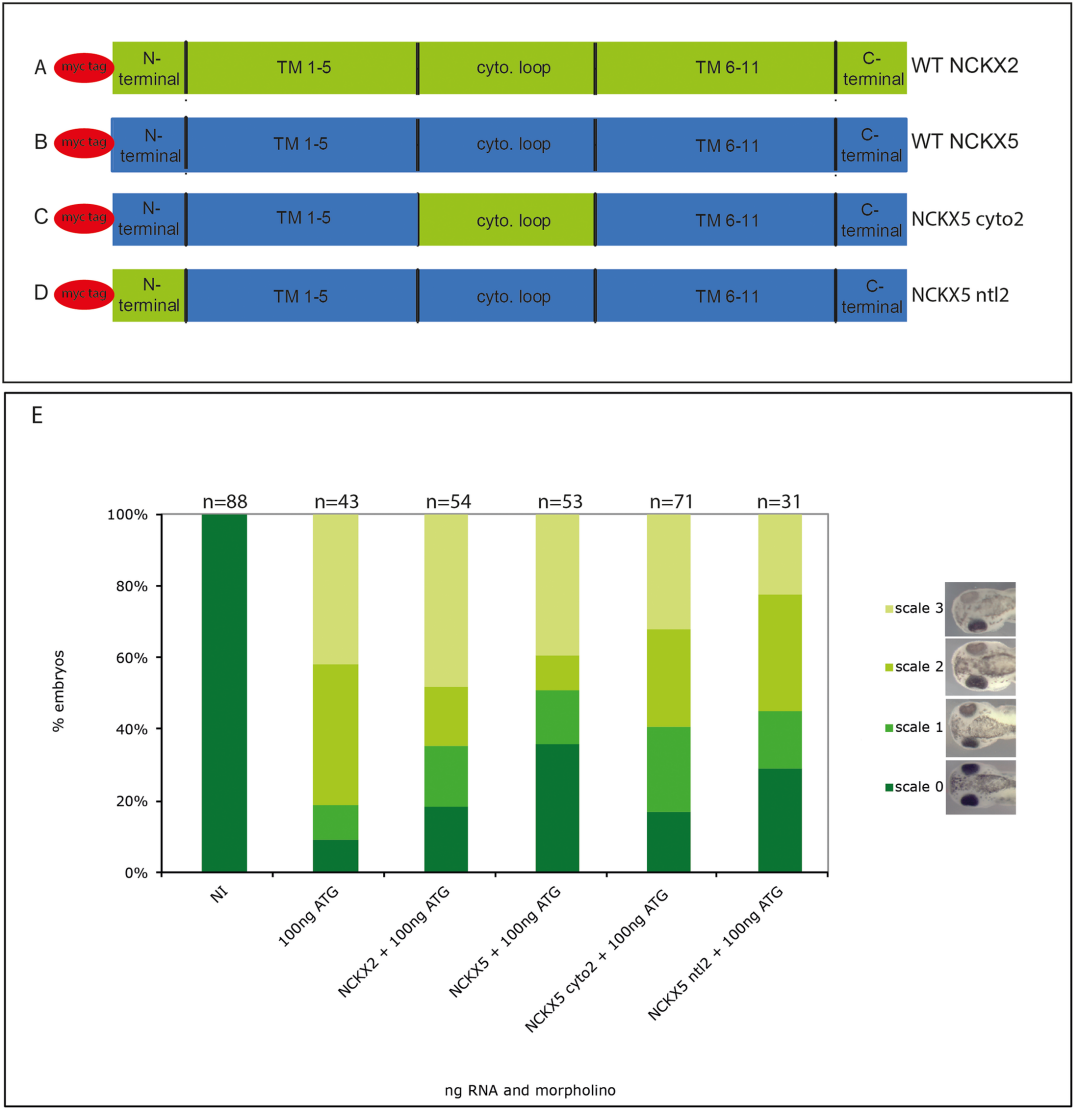
Rescuing the morpholino with chimeric constructs. 5ng RNA of the constructs was co-injected with 100ng morpholino, the embryos were raised to stage 38. The experiments were repeated in triplicate and the results were combined, n values shown at top of graph. (A-D) shows schematics of chimera rescue contructs. (E) Embryos were scored according to the scale in Fig 2. Kruskal Wallis revealed a significant difference between 100ng ATG MO alone and the NCKX5 and NCKX5 ntl2 constructs, p = 0.01 and p = 0.022 respectively.

To look more broadly at which regions of the protein are important for function chimeric constructs of NCKX2/NCKX5 where made. These comprised the N terminal loop of NCKX2 with the rest of NCKX5 (NCKX5 ntl2) and the cytosolic loop of NCKX2 with the rest of NCKX5 (NCKX5 cyto2), (Fig 6). The experimental paradigm was the same as the other rescues.

The NCKX2 and NCKX5 cyto2 chimeric constructs cannot rescue the 100ng ATG morpholino effect, while the NCKX5 and NCKX5 ntl2 constructs can (Fig 6). This is consistent with the above rescue experiments where the wild type construct (effectively the same as NCKX5 here) can rescue the effect of the morpholino. As with the 4C construct, the NCKX5 cyto2 construct cannot provide a rescue. These constructs are both mutants/variants of the large loop.

## Discussion

The ion exchanger; NCKX5, has been shown to be important in pigmentation from fish to humans. Its precise role in this process is still unclear. We have used the frog, *Xenopus laevis* as an *in vivo* tool to investigate the function of NCKX5 in this context.

### *X*.*laevis* SLC24A5 is 70% similar to human SLC24A5

The *X*.*laevis* SLC24A5 encodes a protein of 523 amino acids. When aligned with the SLC24A5 sequence from other species approximately 70% similarity is observed, although there are some differences at the 5’ region between all species. As this region is thought to contain a cleavable signal peptide, it is likely that it is not crucial to the function of the protein. The human protein sequence contains a ns-SNP at position 111; this SNP has been shown to highly correlate with skin colour. We found that at the equivalent amino acid 111 position of the human protein, an alanine residue is present in both the L and S forms of the *X*.*laevis* protein suggesting it does not carry the ns-SNP observed in humans. This is also true for mouse, zebrafish and *X*.*tropicalis*, as these other species also carry the alanine residue. This residue is found in a highly conserved region of the protein.

### SLC24A5 is exclusively expressed in melanophores

SLC24A5 expression is seen in the epidermal pigment cells and the RPE of the eye. Expression by *in situ* hybridisation is first detected at stage 25, (earlier stages were analysed but no expression was seen, data not shown), in the developing melanophores at the dorsal neural tube and becomes more intense as the embryo develops through stages 30 and onwards, as more pigment cells develop across the embryo, in the lateral pigment stripe and pigmented areas of the tail (dorsal and ventral). The dendritic structure of the melanophores is clear. The SLC24A5 expression pattern mirrors that of DCT, [28], a known melanophore marker, strongly suggesting the SLC24A5 expression is indeed in the melanophores. This expression pattern is consistent with that seen in zebrafish embryos, where both SLC24A5 and DCT are detected in the melanophores and RPE [5]. No expression was detected in other tissues such as pancreas, heart and lung, as seen in the mouse [5].

Our results confirm that unlike other SLC24 genes, SLC24A5 is not expressed in the brain or other organs, and that its expression is quite restricted. Of the NCKX family, NCKX5 is the only protein expressed in non-excitatory tissue i.e. the melanophores, with the exception of a report where NCKX1 was found in platelet cells [29].

### Morpholino knockdown of NCKX5 causes a significant reduction in pigmentation

Morpholino knockdown of NCKX5 in *X*.*laevis* embryos causes a clear reduction in pigmentation. This is most prominent in the RPE of the eye but also noticeable in the lateral pigmentation. The morpholino effect is proportional to the concentration of morpholino injected. The morpholino phenotype described here is consistent with that seen in the zebrafish, where morpholino knockdown of NCKX5 phenocopies the reduction in pigmentation that is characteristic of the golden phenotype [5].

As an ion exchanger protein, NCKX5 is not expected to be directly involved in a signalling pathway. However, Ginger *et al*. (2008) showed that siRNA mediated knockdown of SLC24A5 in human melanocytes resulted in a significant decrease in protein expression of known melanocyte markers Pmel17, MART1, Tyr and Tyrp1, suggesting that SLC24A5 is important throughout melanosome development. In the absence of antibodies, *in situ* hybridisation analysis was used here to detect any changes in the expression of key melanogenesis genes following NCKX5 morpholino knockdown *in vivo*. We show that DCT (and tyrosinase, data not shown) transcript expression is not affected by the NCKX5 morpholino knockdown. These data also show that the melanophores are able to migrate and have normal morphology following morpholino treatment. The presence of DCT and tyrosinase suggests that the melanophores do have the appropriate infrastructure to enable pigment production, but the lack of NCKX5 inhibits this to a degree. Histological analysis of morpholino treated tadpoles shows how severe the pigment effect of the morpholino can be. This is particularly clear in the eye, which while remaining normal in size and shape appears to be lacking a layer of pigment thus giving a lighter phenotype.

### Knockdown of NCKX5 can be rescued by human NCKX5

Currently it has been shown that SLC24A5 is important for pigmentation and that it likely encodes NCKX5; however the ion exchanger activity of this protein has not yet been directly linked to its role in pigmentation. In this study we have used a functional approach to analyse the role of NCKX5 in pigmentation. Mutant human NCKX5 constructs were used to rescue the morpholino induced pigmentation phenotype. These constructs carry mutations at equivalent residues, in NCKX5, known to be important for NCKX2 function, as well as one that carries the ancestral SNP at A111T, and two NCKX2/NCKX5 chimeras. The *X*.*laevis* wild type construct was also used to rescue and to demonstrate the specificity of the morpholino, and two mutants were made in the Xenopus background. These experiments were designed to elucidate key residues for NCKX5 function in melanogenesis. By sequence similarity, it was hypothesised that the human wild type clone should rescue or partially rescue the morpholino phenotype, as was also seen in the zebrafish [5]. The wild type human NCKX5 is 65.8% similar to the *X*.*laevis* sequence, at the amino acid level. In rescue experiments we found it could rescue the morpholino knockdown of *X*.*laevis* NCKX5. This was not as strong a rescue as the *X*.*laevis* clone, which is to be expected. This fits with the zebrafish work, where human SLC24A5 could partially rescue the SLC24A5 morpholino [5].

It was also thought that the A111T mutation might provide a partial rescue, although this may be less significant as the nsSNP causes the protein to be less active [18]. However our data shows that A111T can rescue almost as well as the human wild type, indicating that perhaps this mutation is not so vital in Xenopus pigmentation production. The Xenopus A111T was not available to test.

Based on data from work done with NCKX2 [27, 30] the D383N mutation was not expected to provide a rescue as this protein shows no calcium transport, *in vitro* [27], the Asp residue at this position is conserved in *X*.*laevis* NCKX5 and nearly all other NCKX in the data base. However, the D383N mutant gave a strong rescue, which was confirmed using the Xenopus D389N mutant (Fig 5), suggesting that the calcium transport function of NCKX5 might not be essential for its role in pigmentation, and that the protein may regulate pigment production via another mechanism. To further explore this phenomenon we used another mutant in the Xenopus background, E143Q. E143 is a key Ca^2+^ binding residue, its mutation *in vitro* also abolishes calcium transport, and we find in our rescue paradigm it cannot recover the pigmentation phenotype. These conflicting results are perplexing, it is possible that the D389 residue is less important *in vivo*. The results of a double mutant would be interesting. It should be taken into account that some ion exchangers do have secondary function(s). Walters *et al*. showed that Xenopus NKCC1 (SLC12A2) could induce a secondary axis, independently of its ion exchanger activity [31]. We tested if SLC24A5 could induce a secondary axis but it could not (data not shown). Cysteine residues are known to be important in many proteins, particularly for enzymatic reactions and inter/intra molecular interactions [32]. Despite this critical role in many proteins, it has been shown that cysteine residues are not important for NCKX2 function, [33]. Here, we used the 4C mutant to determine if a cluster of 4 conserved cysteines in the cytosolic loop are important for function of the protein. Additionally chimeric constructs of NCKX2 and NCKX5 were made and used in rescue analysis to test various parts of the proteins. These were; NCKX5 with the large cytosolic loop of NCKX2 and NCKX5 with the signal sequence and N-terminal loop of NCKX2 (as depicted in Fig 6). We show that the 4C cysteine mutant cannot rescue the morpholino knockdown; this suggests that these cysteine residues are important for NCKX5 function in regulation of pigmentation, potentially through metal ion binding however this mechanism is unclear.

As expected the wild type NCKX5 construct can rescue the morpholino effect. The NCKX5 ntl2 chimera can also rescue, this suggests the N-terminal region of NCKX5 is not crucial for its function, or the NCKX2 N-terminal region can substitute its function. NCKX2 was not expected to rescue as although it has the same core function it is normally expressed in different tissues. The lack of rescue from the NCKX5 cyto2 chimera is also consistent with the previous data where the 4C construct could not rescue (Fig 4). The 4C construct has had its loop cysteines replaced with glycine, while the cytosolic loop of NCKX2 has no cysteines in it, so this chimera is comparable with the 4C mutant.

## Conclusions

A role for NCKX5 in pigmentation is now well established, *in vitro* and *in vivo* and across different species. NCKX5 is suggested to be a potassium dependent sodium calcium exchanger, although direct evidence of this is limited, due to the unusual intracellular location of NCKX5. Linking together ion exchange and pigmentation has proved challenging. Previous speculation has indicated a role for NCKX5 in pH maintenance [5], which is known to be important for melanin production [34], however how exactly NCKX5 links to proton exchange has not been determined experimentally. As an important signalling molecule for many processes, Ca^2+^ homeostasis has to be tightly regulated and as a Na^+^/Ca^2+^/K^+^ exchanger it is acceptable to suggest a role for NCXK5 in Ca^2+^ regulation. However, the DN mutant construct has been shown to reduce the exchanger function of this protein, *in vitro*, but yet can rescue the mutant phenotype in the *Xenopus* system. Homo or hetero dimerisation of NCKX5 has not been investigated. Dimer formation may go some way to explain the lack of rescue from the 4C mutant, as these cysteine residues could be important for inter-protein bonding. Our results however, suggest that NCKX5 may regulate pigmentation through a novel mechanism, which has yet to be determined.

## Methods

All experiments were performed in compliance with the relevant laws and institutional guidelines at the University of East Anglia. The research has been approved by the ethics committee of the University of East Anglia.

### PCR and cloning

An internal 800bp fragment of *X*.*laevis* SLC24A5 was generated using forward primer 5’ TCATGGCAATTGGAAGTTCA 3’ and reverse primer 5’ GTTCCAGCAGCGAGTAATG 3’. RNA extracted from 10 stage 38 embryos using the Qiagen RNeasy kit, was used to generate the template cDNA. The PCR was set up as follows; each 50μl reaction contained; 1X PCR buffer, 2.5mM MgCl_2_, 0.2μM of each primer, 0.25mM dNTPs, 1μl cDNA template and 1 unit Taq polymerase and up to final volume of 50μl with dH_2_O. The conditions were; initial denaturation step of 94°C for 3 minutes, followed by 30 cycles of 94°C 30 seconds, 50°C 45 seconds, 72°C 1 minute, the reaction was finished with a 10 minute 72°C step.

This fragment was then used to design primers for RACE PCR to obtain the 5’ and 3’ sequence. The Invitrogen GeneRacer kit was used following instructions. 5’ RACE was set up as above, with 5’ CCAACAACAGGCGGCACATATCCCAAGA 3’ as the 3’ primer and the GeneRacer 5’ primer provided with the kit, touchdown conditions were used, 94°C for 3 minutes, followed by 5 cycles of 94°C for 30 seconds and 72°C for 1 minute, then 5 cycles of 94°C for 30 seconds and 70°C for 1 minute, then 25 cycles of 94°C for 30 seconds, 66°C for 30 seconds and 70°C for 1 minute, the reaction was completed with 70°C for 10 minutes. For 3’ RACE primers; ACCCTCTAATAACCCGCCAAGTGT and the adapter primer provided in the kit were used. The conditions used were as follows; 94°C for 3 minutes, then 30 cycles of 94°C for 30 seconds, 58°C for 45 seconds and 72°C for 2 minutes, and completed with 72°C for 10 minutes.

Primers were designed from the 5’ and 3’ RACE products and used to generate the full sequence of SLC24A5. Stage 38 cDNA was used as the template, 12.5μl of BioTaq Premix was used with 1μl template, 0.2μM of each primer (5’ CTGGCAGGGAGAGCAGAGTC 3’ and 5’ TTAGTCACCACACAGTACCATAGG 3’) and made up to 25μl with water. The reaction conditions were 94°C for 3 minutes, then 35 cycles of 95°C for 30 seconds, 64°C for 1 minute, 72°C for 2 minutes, and finished with 72°C for 10 minutes.

All PCR products were TA cloned into pGEMTeasy vector and sequenced.

### In situ

Whole mount in situ hybridisation was carried out as previously described [25]. Sense and anti sense probes were generated. For histological examination embryos were cyrosectioned as previously described [35], following embedding in OCT, samples were snap frozen on dry ice and stored at -20°C

### Embryo microinjection

*X*.*laevis* eggs were obtained manually and fertilised *in vitro*, adult males were sacrificed by schedule 1 method in line with the UK Animals (Scientific Procedures) Act, 1986. Following de-jellying in 2% cysteine embryos were kept in 0.1XMMR at 12°C before microinjection. Microinjection was performed in 3% Ficoll, at 20°C, embryos were injected into 1 cell at the 2 cell stage, in the animal pole. Embryos were left to develop at 22°C and fixed at stage 38 for 1 hour in MEMFA.

Capped RNA for injections was prepared using the SP6 mMESSAGE mMACHINE kit. 5ng of cRNA was injected per embryo alone or with morpholino. Antisense morpholinos were designed and provided by GeneTools, these were tested in vitro using the Promega TNT coupled reticulocyte lysate system. The morpholino to the ATG start site of *X*.*laevis* SLC24A5 was TCAGAGCAACAGAACCTTTCTCCAT and scrambled control morpholino was also provided, CCTCTTACCTCAGTTACAATTTATA.

### Rescue experiments

All mutant constructs were myc tagged. Capped RNA was synthesised from the rescue constructs, 5ng of this was co-injected with 100ng of morpholino, again into one cell at the two cell stage. A mutant *X*.*laevis* construct was generated such that the morpholino would not recognise it, the original sequence ATGGAGAAAGGTTCTGTTGCT was converted to ATGGAGAAAGGaTCcGTTGCT.

### Western blotting

At stage 11, 10 embryos from each injection were lysed in 100μl NP40 containing protease inhibitors and left on ice for 10 minutes. To extract the proteins 300μl of Freon was added, the mixture was vortexed then spun at 4°C, at full speed for 15 minutes. After centrifugation the top layer of the mixture was removed and stored at -20°C. Anti Myc antibody was purchased from Abcam (9B11), secondary antibody was anti mouse conjugated to HRP.

## Supporting information

S1 Fig Alignment of full length *X*.*laevis* SLC24A5 with other species. The A111T NS-SNP is highlighted by the yellow box. HS; homo sapiens, MM; *mus musculus*, ZF; zebrafish, XT; *Xenopus tropicalis*, XL; *Xenopus laevis*.
(TIF)

S2 Fig In vitro translation analysis of ATG morpholino efficacy. A. The control luciferase is not affected by the morpholino. SLC24A5 protein expression is significantly reduced in the presence of the ATG morpholino, even at low concentrations. A control morpholino (CMO) does not effect SLC24A5 translation. B. Both the long and short alleles of SLC24a5 have the same sequence around the translation start site. The Morpholino is therefore predicted to knockdown both forms.
(TIF)

S3 Fig Western blot analysis of rescue constructs from embryo lysates following injection of cRNA. The rescue samples can be detected by their myc tag using a myc antibody. The full length protein is 43kDa, as marked by arrow. HS+MO sample shows the human sequence is not targeted by the *X*.*laevis* morpholino. NI; non injected, WT; wild type, AT; A111T, DN; D383N; cytosolic loop mutant, 4C; cysteine mutant, XL; *X*.*laevis*, WT+MO; human wild type co-injected with Morpholino.
(TIF)

S4 Fig Splice target morpholino results. Splice targeted morpholino to SLC24A5 results in a reduction of pigmentation, similar to that seen by the ATG morpholino. Embryos were scored according to the scale presented.
(TIF)
