# Supplementary figures and images for: A functional approach to understanding the role of NCKX5 in Xenopus pigmentation
Source: PLoS One. 2017 Jul 10;12(7):e0180465. doi: 10.1371/journal.pone.0180465 (PMC5503238; doi:10.1371/journal.pone.0180465)

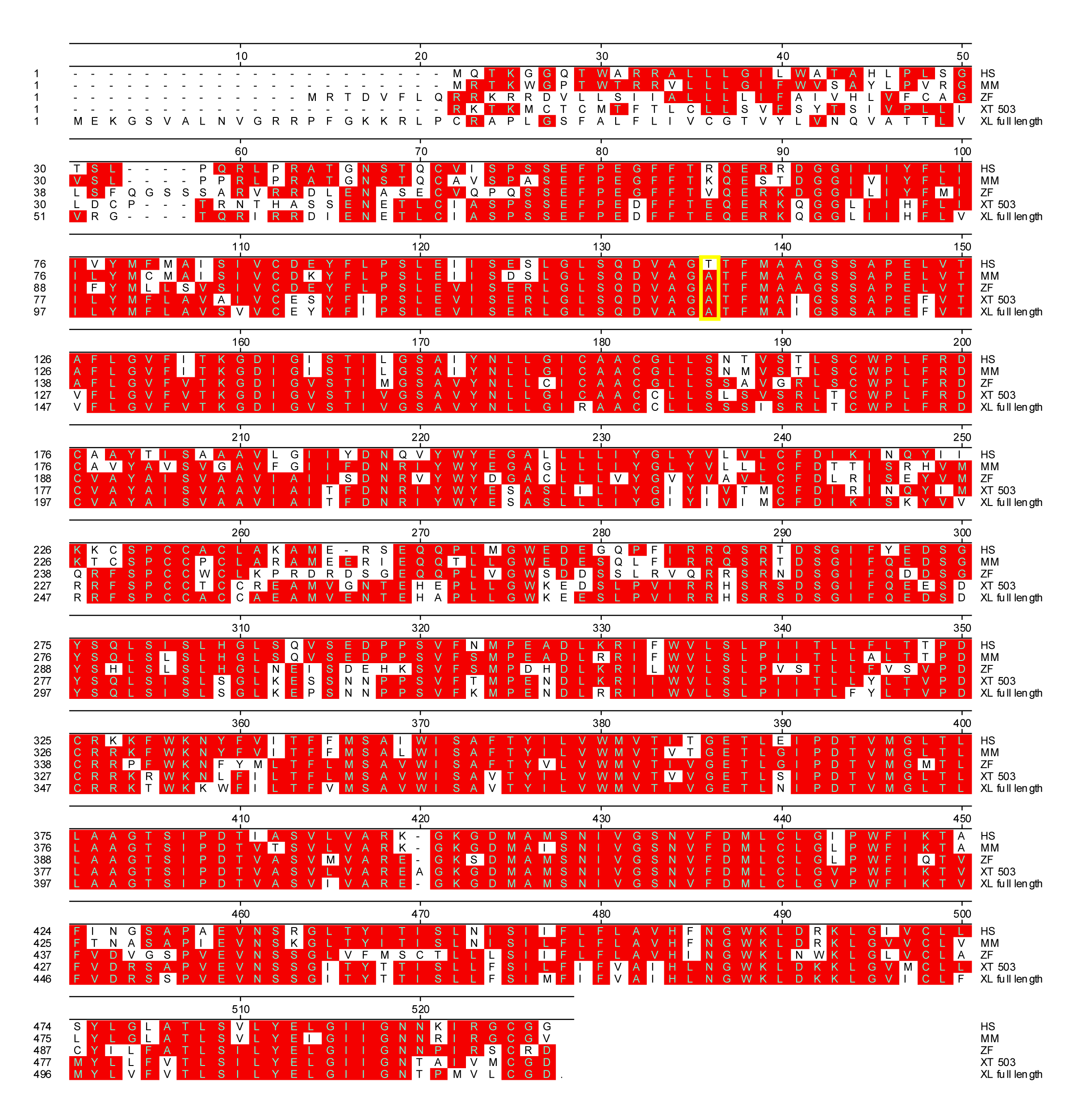

Supplement: S1 Fig — The A111T NS-SNP is highlighted by the yellow box. HS; homo sapiens, MM; mus musculus, ZF; zebrafish, XT; Xenopus tropicalis, XL; Xenopus laevis. (TIF) [file pone.0180465.s001.tif]

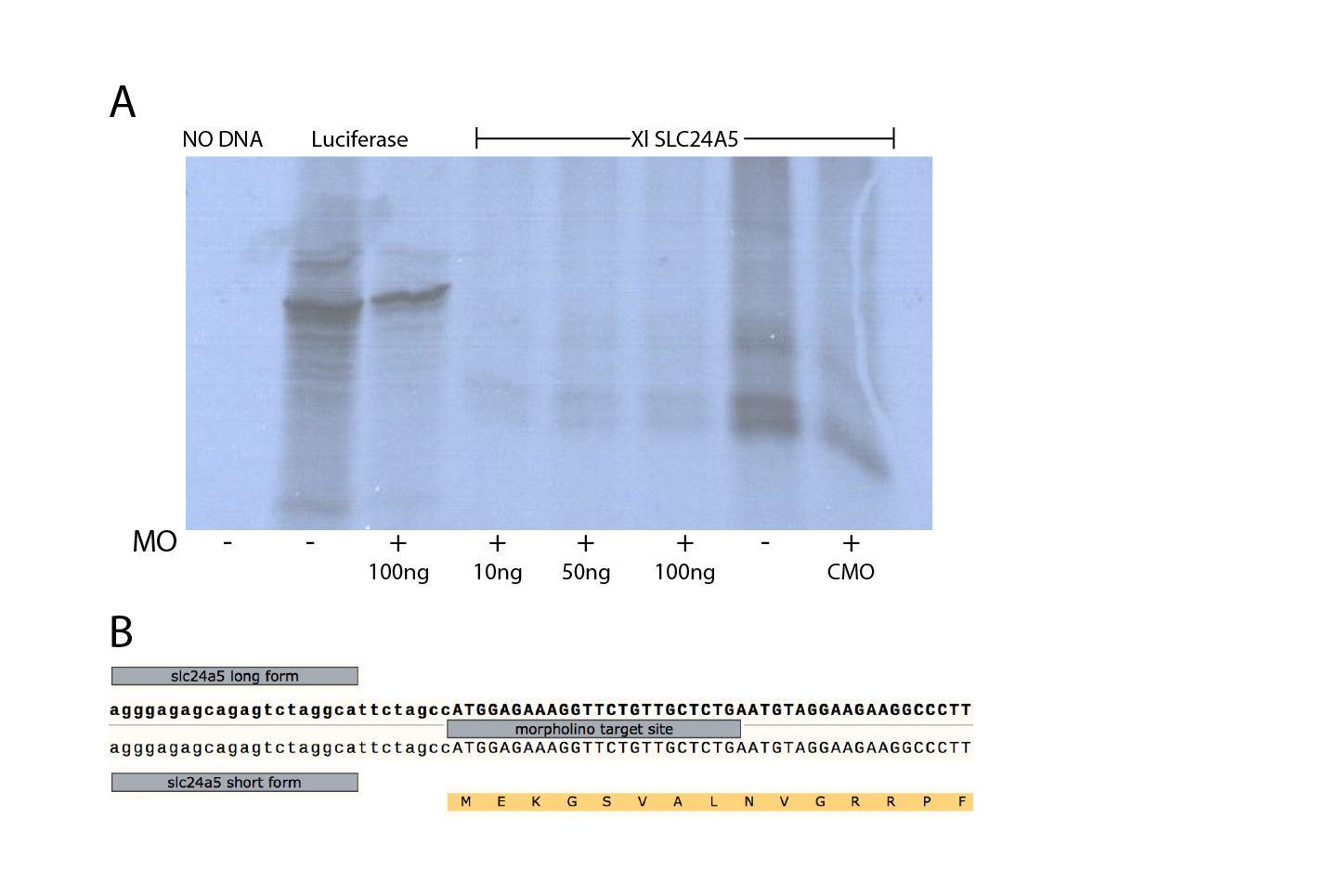

Supplement: S2 Fig — A. The control luciferase is not affected by the morpholino. SLC24A5 protein expression is significantly reduced in the presence of the ATG morpholino, even at low concentrations. A control morpholino (CMO) does not effect SLC24A5 translation. B. Both the long and short alleles of SLC24a5 have the same sequence around the translation start site. The Morpholino is therefore predicted to knockdown both forms. (TIF) [file pone.0180465.s002.tif]

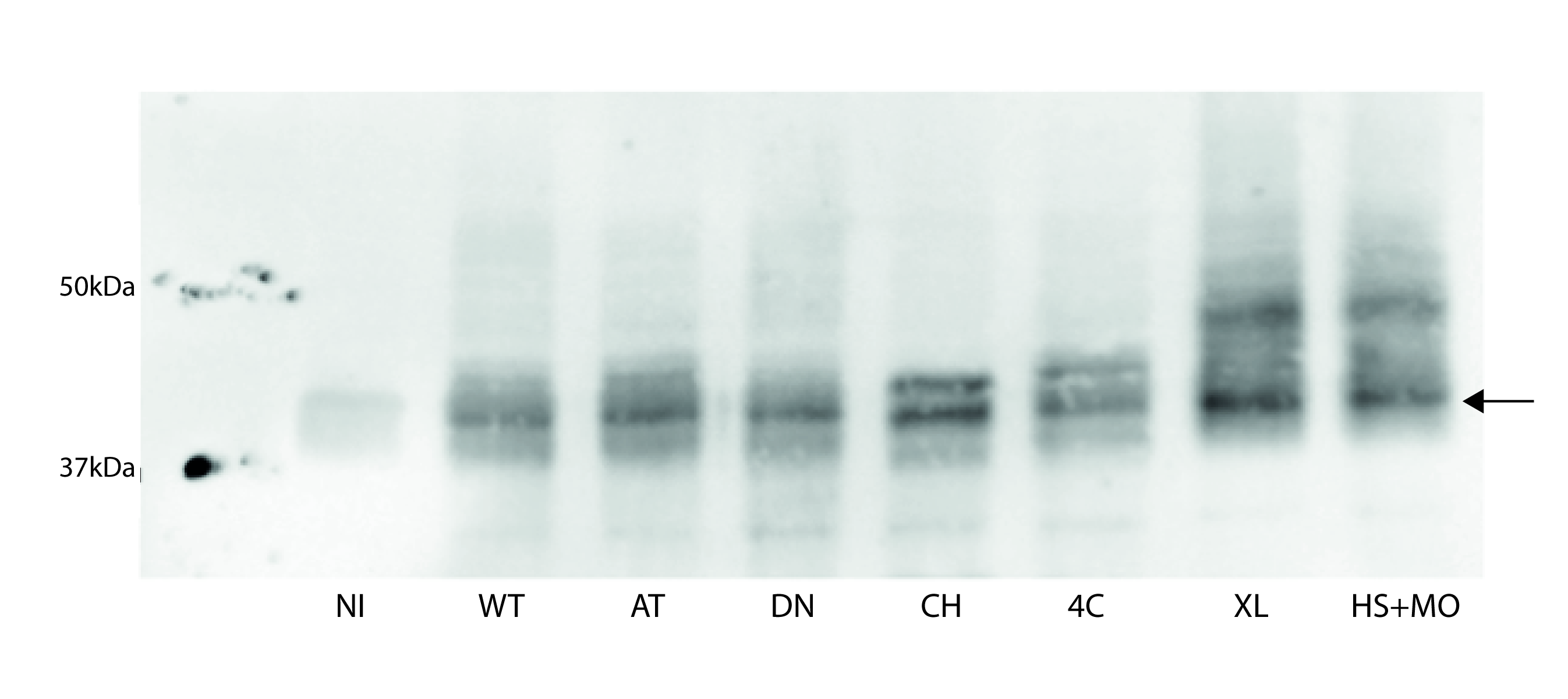

Supplement: S3 Fig — The rescue samples can be detected by their myc tag using a myc antibody. The full length protein is 43kDa, as marked by arrow. HS+MO sample shows the human sequence is not targeted by the X.laevis morpholino. NI; non injected, WT; wild type, AT; A111T, DN; D383N; cytosolic loop mutant, 4C; cysteine mutant, XL; X.laevis, WT+MO; human wild type co-injected with Morpholino. (TIF) [file pone.0180465.s003.tif]

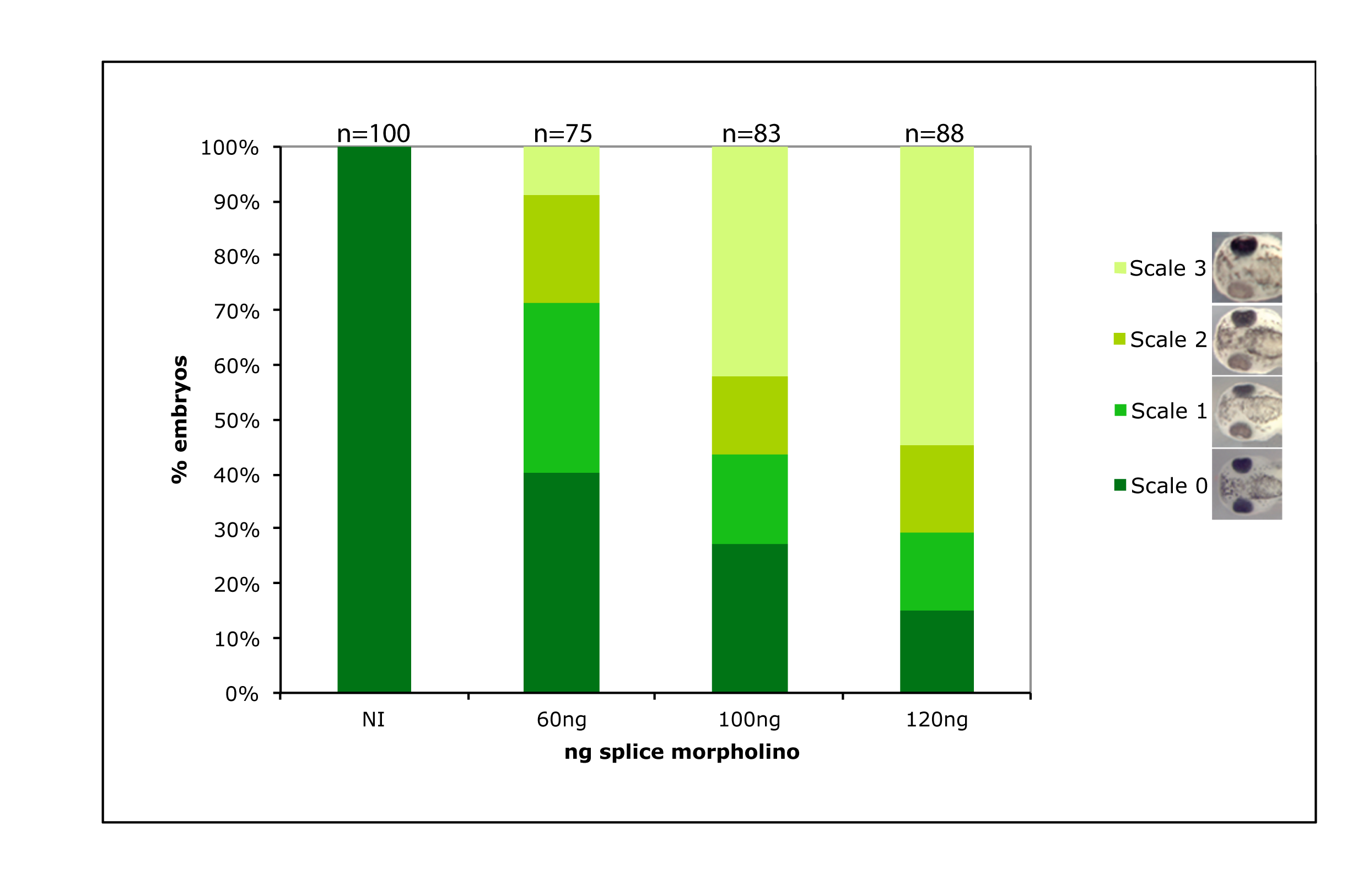

Supplement: S4 Fig — Splice targeted morpholino to SLC24A5 results in a reduction of pigmentation, similar to that seen by the ATG morpholino. Embryos were scored according to the scale presented. (TIF) [file pone.0180465.s004.tif]
